# Systematizing Effective Practice, Embedding It in Standard Practice

**DOI:** 10.1016/j.patter.2020.100151

**Published:** 2020-11-13

**Authors:** The UK Reproducibility Network Steering Group

**Affiliations:** 1 https://www.ukrn.org/

## Abstract

Academia uses methods and techniques that are cutting edge and constantly evolving, while the underlying cultures and working practices remain rooted in the 19^th^-century model of the independent scientist. Standardization in processes and data standards—delivered via foundational and ongoing training—could ensure a common minimum standard, increase interoperability across the sector, and drive improvements in research quality. But change will require a coordinated approach that recognizes the systems nature of the challenge.

## Main Text

Academia represents a paradox. On the one hand, many of the methods and techniques used across disciplines are cutting edge and constantly evolving. However, at the same time, our underlying cultures and working practices remain rooted in the 19^th^-century model of the independent scientist. Research groups are effectively small, artisanal businesses, each crafting outputs—often exquisite, but the product of the unique skills and processes of that group. Is there room for improvement?

Certainly, there is scope for a more coordinated approach. For example, a degree of standardization in processes and data standards—delivered via foundational and ongoing training—could serve to ensure a common minimum standard, as well as increasing interoperability across the sector. This, in turn, could drive improvements in research quality. But change will require a coordinated approach that recognizes the systems nature of the challenge.

### Systematizing Effective Practice

What skills are lacking, or exist but are not standardized across the sector? This depends very much on the specific disciplines. For example, in genomics the use of common genotyping chips and imputation platforms enforces a degree of standardization on the data generated and enhances interoperability. Indeed, the widespread availability of summary data from genome-wide association studies, all in a (broadly) common format, has supported the growth of entire methodologies that rely largely or entirely on these data.^1^ However, even here there is room for improvement—data are not shared as commonly as they could be,^2^ and notation remains non-standard or ambiguous in some cases,^3^ which leads to the potential for error.^4^

In other disciplines, however, including many laboratory disciplines (*in vitro*, *in vivo*, animal, human, etc.) there may be approaches considered standard within a group or laboratory but not across the discipline. Delivering training in basic skills—from file-naming conventions to data curation (including formatting of data files, the use of data dictionaries and codebooks, the need for metadata, the importance of using non-proprietary formats, and other principles of FAIR data,^5^ etc.) could go a long way to creating common, sector-wide standards in quantitative disciplines. This training could be built into undergraduate and postgraduate training, not least because these represent key transferable skills relevant to a wide range of careers and industries.

These foundational skills could be considered the base from which to drive forward research quality. This might include training in more discipline-specific methodological skills, and again this would benefit from a degree of standardization of expectations. Here, reporting guidelines may provide a valuable framework. While these are generally intended to describe aspects of a study that should be reported, the importance of many items included arises from their centrality in assessing the validity of a research claim, and thus they speak to important aspects of research design, conduct, and analysis as well as to research reporting. The EQUATOR network (https://www.equator-network.org) provides reporting guidelines for a range of methodologies across biomedicine—from preclinical animal studies to randomized controlled trials. They therefore provide a framework for methodological training in disciplines where an appropriate guideline exists.

Reporting guidelines are far from perfect—there remain gaps in the coverage as well as duplication (where multiple guidelines exist for similar study designs). Nevertheless, they represent an opportunity for developing a more coordinated approach. They also illustrate the systems nature of the problem. At present, these guidelines are mandated or encouraged by many (but not all) journals, but again focus on the reporting stage when they could perhaps more valuably be incorporated at the study design stage. And this suggests a role for funders, by requiring something similar at the grant submission stage, as well as a role for institutions in terms of the training they offer at undergraduate and postgraduate levels (and beyond).

### Embedding Effective Practice as Standard Practice

Delivering the highest standards in research is not achieved or sustained without considerable effort. As with many other aspects of our lives, the last 40 years has seen remarkable growth in the measurement of academic performance, ostensibly to improve that performance. Surrogate outcomes and metrics have been given a prominent and critical role. A tool that was originally developed to help librarians choose journal subscriptions—the journal impact factor—was repurposed to evaluate individual research outputs and the performance of entire institutions and to inform funding decisions for both. Individual researchers have left the profession, and entire departments have been closed, because of a lack of perceived success under the prevailing “rules of the game.”

Incentive structures are critical; they must be *appropriate* and in turn allow us to properly evaluate performance without distorting the very thing we seek to improve. Evaluation needs to be rigorous, transparent, multidimensional, and resistant to “gaming” (i.e., manipulations intended to improve performance scores, rather than underlying performance). At present, quality evaluations are too blunt, too infrequent, and too unwieldy to provide focused motivation to drive improvement. Many—for example the UK Research Excellence Framework—require considerable time and effort in their preparation and assessment. Moreover, the imprecision in these evaluations encourages “gaming.”

So how might we provide incentives to systematizing effective practice? The conventional approach of “quality through evaluation” could play a central role, but this would require that that any evaluation be richer, deeper, and timelier than is currently the case. The UK Audit Commission has suggested that the desired characteristics of performance measures include that they should avoid perverse incentives and be relevant, clearly defined, verifiable, cost effective, and statistically valid.^6^ Our measures of performance should manifest as many of these characteristics as possible.

At present they do not. In certain fields where there is consensus around markers of quality, it is possible to use text-mining approaches to evaluate reporting of key methodological variables.^7^ This would complement existing approaches and these tools, and their reporting, might usefully be developed further. In fields where there is no community-derived consensus for markers of methodological quality, this raises the slightly awkward question of what it is that is being measured at present and provides a strong motivation for the development of such consensus.

### Conclusion

An alternative to “quality through evaluation” is “quality by design.” If an institution or research group has robust systems in place to manage research quality, then it seems likely that quality will improve more rapidly and more sustainably than research quality for an institution or research group that does not. Moreover, if those systems are shared across institutions, this will not only ensure that quality will improve across those institutions but also that this approach will improve interoperability across those institutions, making it easier, for example, for early-career researchers to move seamlessly between them.

Moreover, while it “seems likely” that these approaches will have the desired effects, this remains a series of hypotheses rather than a set of interventions supported by robust evidence. While increasing incentives for institutions to strengthen their efforts in research improvement in general is desirable, more prescriptive requirements or measures of performance need to be supported by evidence that they influence, or measure, fundamental aspects of research quality rather than epiphenomena susceptible to gaming. Again, working across multiple institutions will provide a platform for more robust evaluation as well as benchmarking. Quality is not zero sum—a rising tide lifts all boats.

Training in foundational skills—and in particular, data skills—remains at the heart of this. Many researchers lack basic formal training in file-naming conventions, version control, and data management. Where training exists, it is often *ad hoc* and specific to a particular research group, leading to friction and inefficiency when researchers move between groups and institutions. Although institutions may offer relevant training, attendance is often not incentivized and is typically focused on early-career researchers when in fact it is mid-career and senior researchers whose skills are most in need of updating. A systems perspective is necessary, to ensure that training exists and is incentivized and rewarded.

Focusing on data skills also represents an opportunity to foster multidisciplinarity. While the implicit focus of the examples we have discussed is on the life and biomedical sciences, there are many quantitative disciplines where similar issues apply, and many other disciplines—for example, in qualitative research and in the arts and humanities—where quite different issues relating to data exist. These include interesting conversations about what constitutes data across a range of disciplines and what common solutions might be found—as well as which cases require specific solutions. Primary resource texts, archives, and representations of art are all forms of (often digital) data.^8^

**Figure 1:**
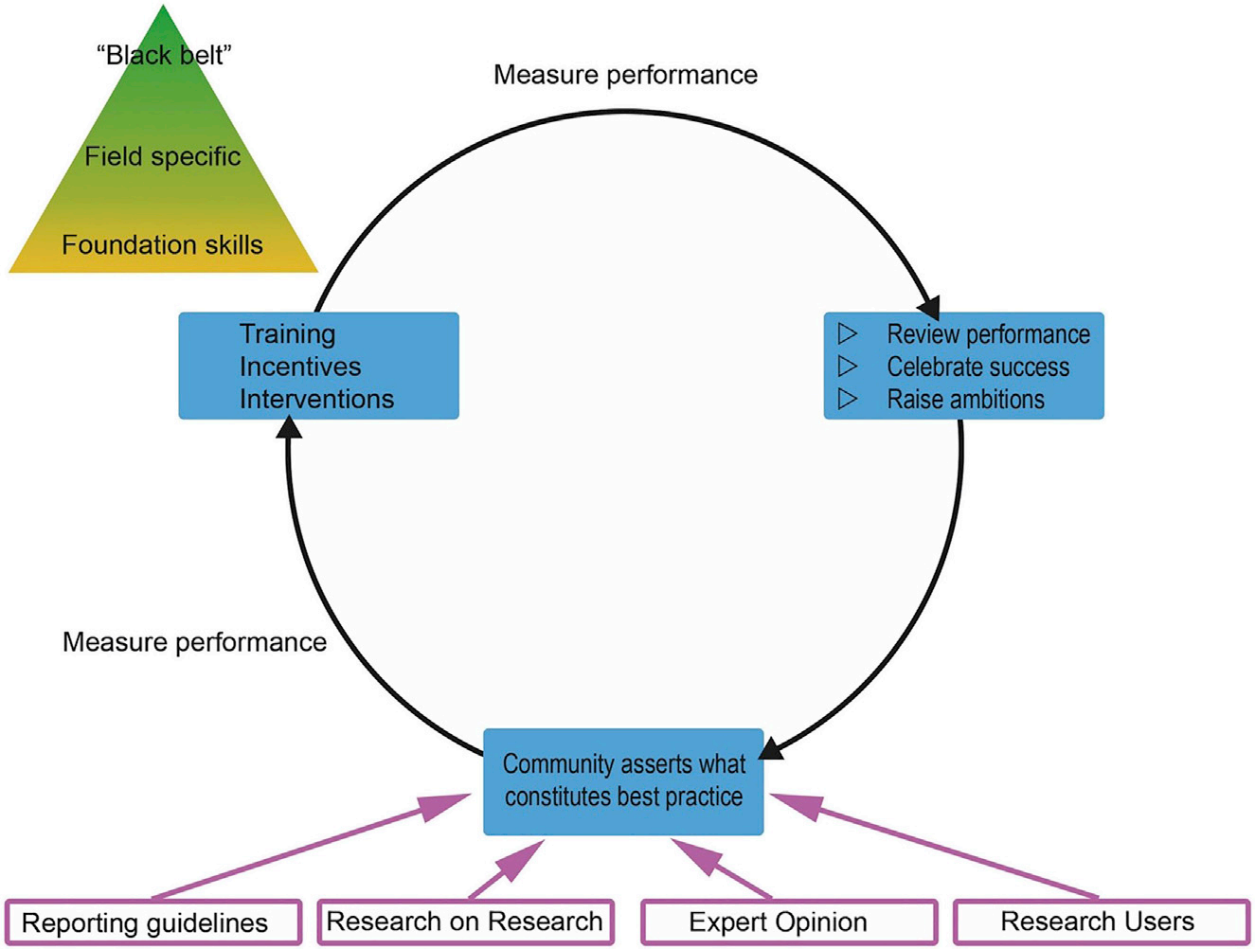
Establishing a Cycle of Continuous Improvement

Effective change will require a coordinated approach, involving researchers themselves, institutions, funders, publishers, learned societies, and other sectoral organizations.^9^ By developing a consensus across this community regarding what constitutes effective practice, we can launch a cycle of continuous improvement (Figure 1), with training, incentives, and interventions to improve standards, complemented and reinforced by reviews of performance as well as the celebration of success and a general culture of raised ambitions. Ongoing evaluation—research on research, the development and refinement of reporting guidelines, and a culture of engagement across the community—and in particular with research users themselves, will lead to real change.
